# Static Attitude Determination Using Convolutional Neural Networks

**DOI:** 10.3390/s21196419

**Published:** 2021-09-26

**Authors:** Guilherme Henrique dos Santos, Laio Oriel Seman, Eduardo Augusto Bezerra, Valderi Reis Quietinho Leithardt, André Sales Mendes, Stéfano Frizzo Stefenon

**Affiliations:** 1 Department of Electrical Engineering, Federal University of Santa Catarina, Florianópolis 88040-900, Brazil; guilherme.dos.santos@spacelab.ufsc.br (G.H.d.S.); eduardo.bezerra@ufsc.br (E.A.B.); 2 Graduate Program in Applied Computer Science, University of Vale do Itajaí, Itajaí 88302-901, Brazil; 3 VALORIZA, Research Center for Endogenous Resources Valorization, Instituto Politécnico de Portalegre, 7300-555 Portalegre, Portugal; valderi@ipportalegre.pt; 4 Expert Systems and Applications Lab, Faculty of Science, University of Salamanca, Plaza de los Caídos s/n, 37008 Salamanca, Spain; andremendes@usal.es; 5 Faculty of Engineering and Applied Science, University of Regina, Regina, SK 3737, Canada; sfc079@uregina.ca

**Keywords:** attitude determination, machine learning, neural network, measurement uncertainty

## Abstract

The need to estimate the orientation between frames of reference is crucial in spacecraft navigation. Robust algorithms for this type of problem have been built by following algebraic approaches, but data-driven solutions are becoming more appealing due to their stochastic nature. Hence, an approach based on convolutional neural networks in order to deal with measurement uncertainty in static attitude determination problems is proposed in this paper. PointNet models were trained with different datasets containing different numbers of observation vectors that were used to build attitude profile matrices, which were the inputs of the system. The uncertainty of measurements in the test scenarios was taken into consideration when choosing the best model. The proposed model, which used convolutional neural networks, proved to be less sensitive to higher noise than traditional algorithms, such as singular value decomposition (SVD), the q-method, the quaternion estimator (QUEST), and the second estimator of the optimal quaternion (ESOQ2).

## 1. Introduction

The process of finding rotations between frames—although classic—is recurrent. It consists of providing information about the difference between some objects of interest that are seen from the perspective of the body frame and in the desired frame of reference. This difference is represented as a rotation operation; thus, the goal is to find the rotational axis and rotational angle that best rotate a frame given by ($\vec{a}$, $\vec{b}$, and $\vec{c}$) to another frame given by ($\vec{x}$, $\vec{y}$, and $\vec{z}$). Finding the rotation between frames is applied in many fields, such as robotics [1,2], navigation and control [3], and computer graphics [4,5], among others [6].

In spacecraft navigation, this process is called attitude determination, and typically, various sensors are used to collect information about the objects of interest. These data are returned as a vector, which is known as a measurement or observation vector. It is possible to obtain two orientation parameters from a given measurement vector. However, three orientation parameters are necessary in order to determine an attitude. This leads to an underdetermined problem if only one vector (N=1) is used, since the number of parameters is less than the minimum number that is required. On the other hand, the problem will be overdetermined if two measurement vectors or more (N≥2) are used, since four orientation parameters will be used, i.e., there are too many variables.

Following this reasoning, some measurements are needed to determine a proper attitude such that 1<N<2. However, this is a problem because it is not possible to obtain a non-natural number of measurements; thus, a set of measurement vectors are needed in order to estimate the proper orientation. The first proposed solution was called Wahba’s problem [7], a least-square approximation given by:

(1) $$L\left(A\right)=\frac{1}{2}\sum_{i=1}^{n}a_{i}{∥\vec{b_{i}}-A\vec{r_{i}}∥}^{2},\;\begin{array}{c} \mathrm{such}\mathrm{that} \end{array}\;n\geq 2$$

where $\vec{b_{i}}$ is an observation vector in the body frame, $\vec{r_{i}}$ is a vector in the reference frame, $a_{i}$ is a weight value for each observation vector, and A is the optimal attitude matrix. This first approach to attitude determination led to the development of several new algorithms derived directly from Equation (1), resulting in a trade-off, as more robust models are slower to converge, requiring more computational effort, and very fast algorithms may result in low accuracy [8], thus requiring careful fine-tuning.

To solve the problem, it is possible to use a combination of artificial neural networks (ANNs) with Kalman filters (KFs) [9] or even recurrent neural networks (RNNs) in order to estimate the new attitude [10]. These approaches require some previous attitude information in order to adjust the predictions, that is, they depend on time information. Therefore, these types of algorithms are known as attitude estimation or dynamic attitude determination algorithms. This paper focuses on another type of algorithm that does not need previous attitude information for the prediction, resulting in a static attitude determination.

The quaternion estimator (QUEST) method [11,12], singular value decomposition (SVD)-based method [13], second estimator of the optimal quaternion (ESOQ2) method [14], and q-method [15,16] belong to the static attitude determination class of algorithms, since their inputs are observation vectors that are taken simultaneously or close enough in time that they can ignore spacecraft motion between the measurements. Therefore, they can determine the attitude at that given instant. Conversely, the elimination of the time variable makes this class of algorithms susceptible to the quality of the input data, i.e., the precision of the sensors drastically affects the caliber of the results. Due to their nature, they have a crucial role in the “lost-in-space” scenario [17], where the spacecraft has no previous information about its attitude. Therefore, the navigation system must instantaneously infer an orientation by relying on the quality of measurements taken by its sensors, since this directly affects the performance of the algorithms [18].

In this context, this paper focuses on applying machine learning to the mitigation of the impact of the measurements’ perturbations on the predictions. Specifically, this paper proposes a neural-network-based approach to determining the rotation difference between two coordinate frames based on a convolutional neural network (CNN) architecture. The contribution of this paper is based on the strategy of choosing the model by considering its uncertainty. Furthermore, this paper will show how the model’s performance compares with that of traditional algorithms and if this data-driven approach to static attitude determination can mitigate the impact of input distortion.

## 2. Background

### 2.1. Attitude Representations

Rotation matrices can be parametrized in many different ways, and the most commonly used tools are the Euler axis/angle, Euler angles, and quaternions. The Euler axis/angle describes a rotation as a unit vector $\vec{e}$ that lies along the rotation axis and a rotation angle ϕ about this axis. Although it has a clear physical interpretation, its axis is undefined for ϕ={0,2π}, that is, it is not a continuous representation.

Euler angles are defined as a set of three parameters: ϕ, θ, and ψ. These angles break a single rotation into a sequence of three rotations using two intermediate frames throughout the process. Thus, it is relatively easy to interpret the problem. However, for $\theta =\pm 90^{\circ }$, the solution degenerates, leading to a problem called “gimbal lock”, which causes ϕ and θ to move in the same fashion. That is why one should be careful when using Euler angle representations.

Quaternions are four-dimensional vectors that embed the rotation axis and the rotation angle in a complex domain. Despite not having an apparent physical interpretation, they are the preferable form of parametrization for specialists, since they do not require trigonometric functions and avoid the “gimbal lock” issue. However, this type of representation has an interesting property where $\vec{q}=-\vec{q}$, that is, the antipodes represent the same rotation. Therefore, they are not a continuous representation.

In trying to find a valid continuous representation for SO(3), a new method was proposed by [19]. They assumed that a representation space R produced by a neural network could be mapped into the original space X∈SO(3) through a function f:R→X and mapped back through a function g:X→R.

The function f is a mathematical function that is used as part of the forward pass of the network at both the training and inference time. For 6D representations, this function is guaranteed to return an orthogonal 3 × 3 matrix, and the loss function can then be applied to it. This mapping function is defined as follows for a 6D representation:

(2a) $$\begin{array}{c} f\left(\left[\begin{array}{cc} | & |\\ \vec{a_{1}}\vec{a_{2}}\\ | & | \end{array}\right]\right)=\left[\begin{array}{ccc} | & | & |\\ \vec{b_{1}}\vec{b_{2}}\vec{b_{3}}\\ | & | & | \end{array}\right] \end{array}$$

(2b) $$\begin{array}{c} b_{i}={\left[\left\{\begin{array}{cc} N(\vec{a_{1}}) & \mathrm{if}\;i=1\\ N(\vec{a_{2}}-\left(\vec{b_{1}}\;\cdot \;\vec{a_{2}}\right)\vec{b_{1}}) & \mathrm{if}\;i=2\\ \vec{b_{1}}\times \vec{b_{1}} & \mathrm{if}\;i=3 \end{array}\right.\right]}^{T} \end{array}$$

where N(·) stands for the normalization function, × represents the vector cross product, · represents the vector dot product, and the column vectors $\vec{a_{1}}$ and $\vec{a_{2}}$ are vectors from R that are estimated by the network.

### 2.2. Related Work

As mentioned before, if a spacecraft needs to know its orientation, some prior information must be gathered by sensors. They return the data as three-dimensional unit vectors representing the pointing direction of an object of interest with respect to the body frame. From these data, it is possible to find a proper rotation that will project the observations onto the target frame. There is an extensive field of study covering regression in rotation representations by using ANNs. In the computer vision field, there is a problem that is similar to the one described before, whose premise is to find the orientation of objects with respect to the camera. This task also shares the same goal as attitude determination, that is, it determines the orientations or poses of objects of interest from one coordinate frame to another. In the context of computer vision, this task is referred to as pose estimation.

Xiang et al. [20] tried to estimate the rigid transformation from the object coordinate frame O to the camera coordinate frame C given an input image. Each image contains a collection of objects, and the main task is to find their poses (location and orientation), which are represented by a 3D rotation R and a 3D translation T parametrized by quaternions. Thus, they can be written as $R(\vec{q})$. They used the mean squared error loss function to minimize the error, where they measured the distance between a set of 3D points x∈O rotated by both the ground-truth rotation $R(\vec{q})$ and the predicted rotation $R(\vec{\hat{q}})$. However, using this approach, the network performed poorly when the rotation angles were close to $0^{\circ }$ and $180^{\circ }$.

Do et al. [21] used an architecture called mask R-CNN, which took an RGB image containing several objects as input and returned their classes, segmentation masks, and bounding boxes. They added a fourth output on top of this architecture, which tried to estimate the orientation and localization of each object. This output layer was a four-dimensional vector, where the first three scalar values represented the rotation and the last one represented the translation. However, instead of using representations, such as Euler angles and quaternions, they made use of Lie algebra so(3) to represent the rotation, since a skew-symmetric matrix of an arbitrary element from so(3) could be mapped to the SO(3) through exponential mapping. Hence, they only needed to regress a vector $\vec{x}\in R^{3}$ to define a proper rotation.

Point clouds can be interpreted as 3D vectors that describe a shape or object in some arbitrary coordinate frame; hence, this type of data can provide a lot of useful information, such as depth, volume, orientation, and location. In trying to explore this type of information, a novel approach [22] was proposed to address the 6D pose estimation problem: Instead of using RGB images, they used point cloud segments as the input to the system. The proposed neural network was an adaptation of the PointNet architecture [23], which takes a set of point clouds as input and returns a rotation matrix that best describes the orientation of the input data.

Those works addressed the 6D pose estimation task by considering different types of input data, such as RGB images and point clouds, as well as distinct attitude representations. Although all of them achieved good results for the task, they presented some limitations. First, their attitude representations were not adequate for the job because they showed discontinuities for certain angles. Second, the input data did not offer clear information about the orientation or relationship between the coordinate systems, thus demanding more effort from the network to detect those features.

In this context, this paper proposes a model that uses a different type of input data, which is denoted as an attitude profile matrix; this embeds the similarity between two coordinate frames and alleviates the model’s effort. In addition, the proposed model uses another type of attitude representation that can be learned and does not suffer from discontinuities.

## 3. Methodology

**Figure 1:**
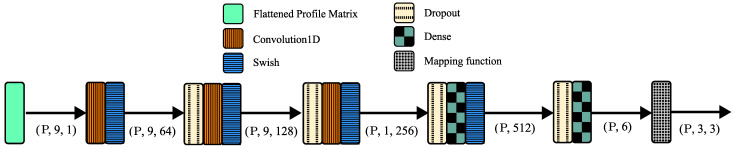
B-Swish (Modified PointNet).

PointNet is a neural network architecture that accepts raw point clouds as input and is robust with respect to input perturbation and corruption [24]. Taking into account the promising results of PointNet, several extensions have been developed [25,26,27,28]. Hence, PointNet was chosen as the base architecture for the experiments presented in this paper. Figure 1 shows the network structure.

Some modifications have been made to the traditional PointNet architecture in order to improve its performance. In the vanilla PointNet, a set of vectors in the original coordinate frame and another set in the target coordinate frame are used as input and fed into the model in pairs. However, in the proposed model, they were replaced by the attitude profile matrix. In fact, the same pairs of vectors were used to build the profile matrix according to Equation (3). By flattening this matrix, an array with the shape (P,9,1) was generated, where P represents the batch size. However, it was considered that all weights $a_{i}$ were equal (star-tracking scenario) because it led to lower errors when compared to random weights, even in the evaluation phase, as can be seen in Section 4.1.

(3) $$B=\sum_{i=1}^{n}a_{i}\vec{b_{i}}{\vec{r_{i}}}^{T}.$$

All one-dimensional convolutions were built using a kernel of size of 9. However, in all but the last convolution, the shape of the feature maps was maintained and, as a result, after applying the convolution operation, the second dimension of the tensor did not change. Next, all rectified linear unit (ReLU) [29] activation functions were replaced with Swish activation [29] because it turned out that ReLU led to more significant errors during training and evaluation. Finally, as the last operation, the mapping function f, which maps a latent rotation representation R to SO(3), was implemented, as performed in [19].

Dropout layers were added after each activation function [30]. The reasons were twofold: to act as a regularizer to prevent overfitting and to measure the model uncertainty. Since the dropout technique randomly drops some neurons with a probability **p** at each forward pass, each update to a layer during training is performed with a different view of the configured layer, forcing the knowledge to be spread out across all neurons equally, thus acting as a regularizer. This can be interpreted as if several neural networks are being trained simultaneously because at each forward pass, the model “changes” its architecture.

Notice that the regular dropout could be interpreted as a Bayesian approximation of a Gaussian process [31]. By applying the dropout during both training and inference, it is possible to analyze it as if predictions from many different networks have been made, that is, a Monte Carlo sample from the space of all possible networks. Hence, it is entitled Monte Carlo Dropout (MCD) [32]. Therefore, a distribution of predictions is gathered and a measure of uncertainty of the model can be evaluated.

In an actual application, the star-tracker algorithm returns two pairs of vectors from a star image: the body vectors, which represent each star in the body frame, and the reference vectors, which represent the same stars collected from the satellite’s onboard database. The proposed model uses those two pairs of vectors to build the attitude profile matrix according to Equation (3). Afterward, the model reads a flattened version of this matrix and estimates the orientation matrix.

### 3.1. Implementation Details

#### 3.1.1. Dataset

The traditional algorithms were designed to determine the best attitude representation given a set of unit observation vectors and their respective weights. In essence, those vectors are random, and the only requirement is that they must be unitary and have a boresight axis, which is the axis that expresses the direction of the observed object.

Following this reasoning, the dataset was built according to the number of samples and the number of observation vectors per sample. First, random unit vectors with arbitrary boresight axes were generated, forming an array with a shape (S,O,3), where S stands for the number of samples, O is the number of observation vectors, and the last dimension is the number of axes of each vector. The last dimension was set to 3, since the data had components in $R^{3}$. The first collections of vectors were chosen to be the reference vectors $\vec{r}$.

The random rotation matrices $A_{\mathrm{true}}$ were generated through Equation (4), where the angles were drawn from a uniform distribution ranging from −π rad to π rad, and the axis vectors were drawn from a uniform distribution.

(4) $$\begin{array}{cc} A & =\left[\begin{array}{ccc} a_{11} & a_{12} & a_{13}\\ a_{21} & a_{22} & a_{23}\\ a_{31} & a_{32} & a_{33} \end{array}\right]\\ a_{11} & =\cos\phi +e_{1}^{2}\left(1-\cos\phi \right)\\ a_{12} & =e_{1}e_{2}\left(1-\cos\phi \right)+e_{3}\sin\phi \\ a_{13} & =e_{1}e_{3}\left(1-\cos\phi \right)-e_{2}\sin\phi \\ a_{21} & =e_{1}e_{2}\left(1-\cos\phi \right)-e_{3}\sin\phi \\ a_{22} & =\cos\phi +e_{2}^{2}\left(1-\cos\phi \right)\\ a_{23} & =e_{2}e_{3}\left(1-\cos\phi \right)+e_{1}\sin\phi \\ a_{31} & =e_{1}e_{3}\left(1-\cos\phi \right)+e_{2}\sin\phi \\ a_{32} & =e_{2}e_{3}\left(1-\cos\phi \right)-e_{1}\sin\phi \\ a_{33} & =\cos\phi +e_{3}^{2}\left(1-\cos\phi \right). \end{array}$$

The random direction cosine matrices (DCM) with shape (S,3,3) served as the ground-truth attitude matrices [33]. The reference vectors were simply rotated by each attitude matrix to generate the body vectors $\vec{b}$ with a shape (S,O,3) as follows:

(5) $$\vec{b_{i}}=\begin{array}{c} A_{\mathrm{true},i} \end{array}\vec{r_{i}}+\vec{n_{i}}$$

where $\vec{n_{i}}$ is a vector of measurement errors drawn from a zero-mean Gaussian distribution $N(0,\sigma_{i})$ with standard deviations $\left\{\sigma_{i}\;|\;10^{-6}\leq \sigma_{i}\leq 0.01\right\}$ serving as additive white Gaussian noise (AWGN) [34].

#### 3.1.2. Training

The dataset was generated with the total number of samples $S=2^{13}$, where 30% was used as the testing dataset and 4% as the validation dataset. The optimizer used was the Adam algorithm [35] with a learning rate of $\mathrm{lr}=10^{-4}$ and a weight decay of $10^{-4}$ being applied every 500 epochs. The lr was also reduced by a factor of ten every 500 epochs.

The work was developed using the Tensorflow 2 framework, and the models were trained on a Nvidia GeForce GTX 1060 6 GB GPU (graphic processing unit). In order to train the network, the geodesic loss function defined by Equation (6) was used to minimize the error. The matrix A is the rotation difference given by the multiplication $A_{\mathrm{true}}^{}A_{\mathrm{pred}}^{T}$. This equation was also used as a metric, along with Wahba’s loss, which is defined by Equation (1), where all weights $a_{i}$ were considered equal during the evaluation. Equation (6) was clipped to lie in the range [−1+eps,1−eps] in order to avoid numerical issues, where $\mathrm{eps}=10^{-7}$ is enough.

(6) $$\phi \left(A\right)=\cos^{-1}\left(\frac{\mathrm{tr}(A)-1}{2}\right).$$

To validate the model’s performance, several training routines were scheduled. For each routine, different values for O were used to check if this could bring a meaningful impact on the model’s robustness. Different models were trained, considering a value for O that ranged from 3 to 7. Every training was performed by considering 2000 epochs with a batch size of 64. In addition, for each O, three different dropout rates were considered: 10%, 15%, and 20%.

## 4. Results

### 4.1. Model Training

Initially, the model was trained with an earlier version of the architecture presented in Section 3, where the pairs of body and reference vectors, which were concatenated along the last dimension, were defined as the input to the network. The number of observation vectors was set as the kernel size parameter in the convolution layers. In all except the last convolution, the shape of the feature maps was maintained. In this assessment, the ReLU activation function was used instead of Swish.

Variations were made to improve the model, since this assessment did not achieve acceptable error values. The pairs of vectors were replaced by the attitude profile matrix, and the architecture presented in Figure 1 was formulated. However, ReLU was considered instead of Swish. Throughout this paper, this model will be called B-ReLU. This model had two versions: B-ReLU-A and B-ReLU-B. The former considered a star-tracking scenario, and the latter considered the actual weights for each pair of inputs when building the attitude profile matrices.

It was possible to notice that using the actual weights to build the B matrix led to poor results; thus, it was decided to use the star-tracking scenario. Since the Swish activation function showed more promising results than the ReLU activation function and its variations, this function was defined as the default in the following analyses. Then, the final model was built (see Section 3), which was called B-Swish.

**Figure 2:**
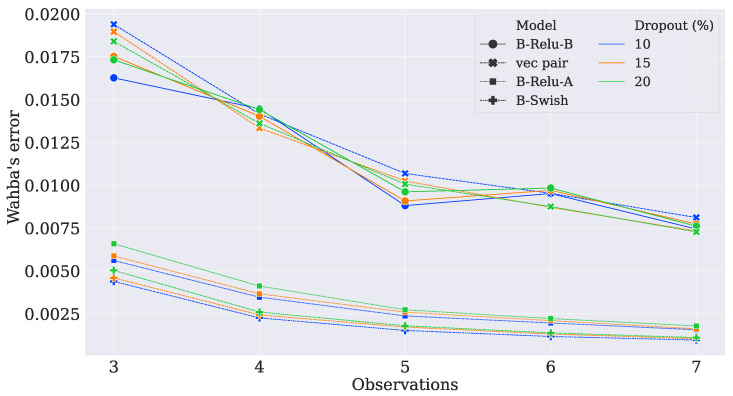
Wahba’s loss.

**Figure 3:**
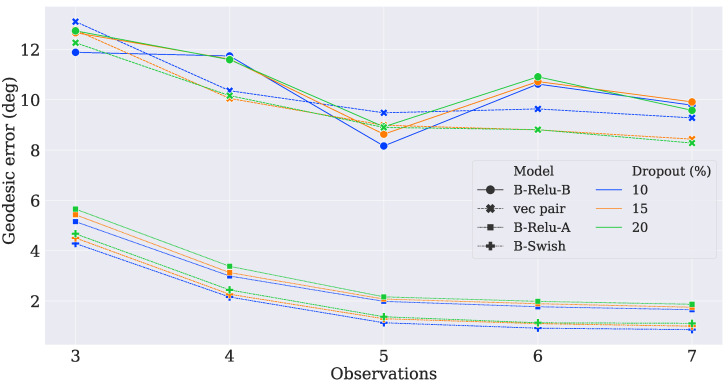
Geodesic error in degrees.

After training the models, their performance was measured by using the validation set. The models whose parameters corresponded to the slightest error in Wahba’s loss were tested. As shown in Figure 2 and Figure 3, the hyperparameter O led to better results on the validation dataset when it was increased. This decrease in error indicated that the network was capturing more insights about the attitude profile matrix because it was using more observation vectors. Moreover, as the dropout rate increased, the network tended to have worse results, indicating that the parameters were over-penalized.

The network struggled to converge when using pairs of body and reference vectors as inputs. Moreover, when using the accurate weights to build the attitude profile, worse results were obtained. The Swish activation function improved the network performance; thus, it was chosen as the final architecture. It was still necessary to verify which of the B-Swish models was more reliable and if the results really reflected the system’s expected behavior when subjected to unseen scenarios.

### 4.2. Model Choice

The models’ performance was verified by using the twelve test cases specified by Markley [36]. Each test case contains a collection of vectors $\vec{r_{i}}$ and their corresponding measurement errors $\sigma_{i}$. These $\vec{r_{i}}$ vectors represent what, in practice, would be the output of the spacecraft’s onboard sensors, such as star trackers, sun sensors, magnetometers, and others. Notice that these data would be returned as three-dimensional vectors. The $\sigma_{i}$ errors represent how accurate the respective observation vectors are, that is, they hold the information about the precision of the sensors that generated these sets of vectors. The body vectors $\vec{b_{i}}$ were constructed by following Equation (5), where $A_{\mathrm{true}}$ is defined as

(7) $$A_{\mathrm{true}}=\left[\begin{array}{ccc} 0.352 & 0.864 & 0.360\\ -0.864 & 0.152 & 0.480\\ 0.360 & -0.480 & 0.800 \end{array}\right].$$

The first four cases consider an ideal scenario, where all observation vectors lie along the boresight axes and are orthogonal to each other. The fifth one demonstrates a case where the vectors are orthogonal but do not lie along the boresight axes. From the sixth scenario onwards, they consider a narrow-field-of-view star tracker, where the rotation about the tracker’s boresight is much less well determined than the pointing of the boresight. Despite the fact that they are handcrafted cases, the tests simulate some interesting scenarios that could happen in an actual application and give an idea about an algorithm’s behavior when subjected to real situations.

The twelve cases were specified as follows:

**Case 1** 
In this case, there are three measurement vectors with measurement noise $\sigma_{1}=\sigma_{2}=\sigma_{3}=10^{-6}$ rad as follows:
  (8) $$\begin{array}{c} \vec{r_{1}}={\left[1,0,0\right]}^{T}\\ \vec{r_{2}}={\left[0,1,0\right]}^{T}\\ \vec{r_{3}}={\left[0,0,1\right]}^{T}. \end{array}$$
**Case 2** 
The same $\vec{r_{1}}$ and $\vec{r_{2}}$ vectors as those from **Case 1** are used with measurement noise $\sigma_{1}=\sigma_{2}=10^{-6}$ rad.
**Case 3** 
The same three vectors from **Case 1** are used, but the noise is increased to $\sigma_{1}=\sigma_{2}=\sigma_{3}=0.01$ rad.
**Case 4** 
The same as **Case 2**, but the the noise is increased to $\sigma_{1}=\sigma_{2}=0.01$ rad.
**Case 5** 
Two reference vectors are used with measurement noise $\sigma_{1}=10^{-6}$ and $\sigma_{2}=0.01$ rad as follows:
  (9) $$\begin{array}{c} \vec{r_{1}}={\left[0.6,0.8,0\right]}^{T}\\ \vec{r_{2}}={\left[0.8,-0.6,0\right]}^{T}. \end{array}$$
**Case 6** 
In this case, there are three measurement vectors with measurement noise $\sigma_{1}=\sigma_{2}=\sigma_{3}=10^{-6}$ rad as follows:
  (10) $$\begin{array}{c} \vec{r_{1}}={\left[1,0,0\right]}^{T}\\ \vec{r_{2}}={\left[1,0.01,0\right]}^{T}\\ \vec{r_{3}}={\left[1,0,0.01\right]}^{T}. \end{array}$$
**Case 7** 
The same $r_{1}$ and $r_{2}$ vectors as those from **Case 6** are used with measurement noise $\sigma_{1}=\sigma_{2}=10^{-6}$ rad.
**Case 8** 
The same three vectors as those from **Case 6** are used, but the noise is increased to $\sigma_{1}=\sigma_{2}=\sigma_{3}=0.01$ rad.
**Case 9** 
The same as in **Case 7**, but the noise is increased to $\sigma_{1}=\sigma_{2}=0.01$ rad.
**Case 10** 
Three reference vectors are used with measurement noise $\sigma_{1}=10^{-6}$ rad and $\sigma_{2}=\sigma_{3}=0.01$ rad as follows:
  (11) $$\begin{array}{c} \vec{r_{1}}={\left[1,0,0\right]}^{T}\\ \vec{r_{2}}={\left[0.96,0.28,0\right]}^{T}\\ \vec{r_{3}}={\left[0.96,0,0.28\right]}^{T}. \end{array}$$
**Case 11** 
The same $\vec{r_{1}}$ and $\vec{r_{2}}$ vectors as those from **Case 10** are used with measurement noise $\sigma_{1}=10^{-6}$ rad and $\sigma_{2}=0.01$ rad.
**Case 12** 
The same $\vec{r_{1}}$ and $\vec{r_{2}}$ vectors as those from **Case 10** are used with measurement noise $\sigma_{1}=0.01$ rad and $\sigma_{2}=10^{-6}$ rad.

For each case, the attitude profile matrix was built as defined in Equation (3) in order to serve as the input to the neural network, where a star-tracking scenario was considered, that is, each observation vector was equally weighted. Then, with the dropout layers enabled, N forward passes were executed through the network to sample a set of possible outputs. In those tests, the definition N=1000 was set.

After each forward pass, Wahba’s error was evaluated by using Equation (1). The weights given by the test case were considered—and are defined in Equation (12b)—and $A_{\mathrm{pred}}$ was considered instead of $A_{\mathrm{true}}$ to measure the true distance. This led to an array of errors with size N for each test case. At the end of the N forward passes, the mean error was measured for each test case, as shown in Table 1. The error values are displayed on a logarithmic scale, where the bold entries represent the smallest values.

(12a) $$\begin{array}{cc} \sigma_{\mathrm{tot}} & ={\left(\sum_{i=1}^{O}\frac{1}{\sigma_{i}^{2}}\right)}^{-1} \end{array}$$

(12b) $$\begin{array}{cc} a_{i} & =\frac{\sigma_{\mathrm{tot}}}{\sigma_{i}^{2}}. \end{array}$$

When choosing the most eligible model, if only the errors displayed in Table 1 were considered, it would still not be possible to notice any meaningful insights. The models were trained using four and five observations, and the use of a dropout rate of 10% was, in general, better than when using other rates. Thus, a procedure described in [37] was followed, which evaluated the rotation error with respect to the average rotation matrix of the predicted DCMs.

Given the set of predicted DCMs $\left\{{\hat{R}}_{j}\;|\;j=1,2,...,N\right\}$, it was possible to calculate an average rotation matrix $R_{\mathrm{avg}}$, that is, a matrix that represented the mean of a distribution of rotation matrices. This average rotation was found as the orthogonal projection of a matrix $\bar{R}=N^{-1}\sum_{j=1}^{N}{\hat{R}}_{j}$ and can be further reviewed in [38], which used an SVD approach in order to evaluate $R_{\mathrm{avg}}$.

Once $R_{\mathrm{avg}}$ was determined, the noise matrices $\Delta R_{j}$ could be evaluated. The new matrices held the orientation differences between each predicted DCM ${\hat{R}}_{j}$ and the average DCM $R_{\mathrm{avg}}$, which could be found by performing the following matrix multiplication:

(13) $$\Delta R_{j}\left(\vec{e_{j}},\phi_{j}\right)=R_{\mathrm{avg}}^{T}{\hat{R}}_{j}$$

where each $\Delta R_{j}$ is parametrized by a rotation axis $\vec{e_{j}}$ and a rotation angle $\phi_{j}$. After finding the noise matrices, their respective Euler vectors $\vec{u_{j}}=\phi_{j}\vec{e_{j}}$ were extracted, and a covariance matrix was calculated as follows:

(14) $$C\left(\vec{u}\right)=\frac{1}{N}\;\left[\vec{u_{1}},\vec{u_{2}},...,\vec{u_{N}}\right]{\left[\vec{u_{1}},\vec{u_{2}},...,\vec{u_{N}}\right]}^{T}.$$

**Figure 4:**
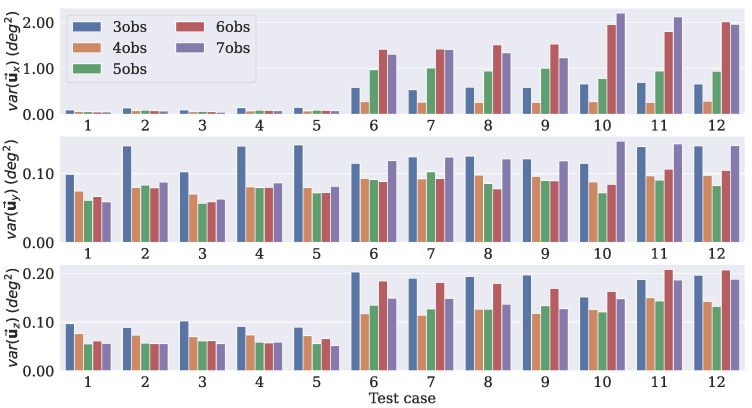
Uncertainties in the axes for each test case considering a dropout rate of 10%.

**Figure 5:**
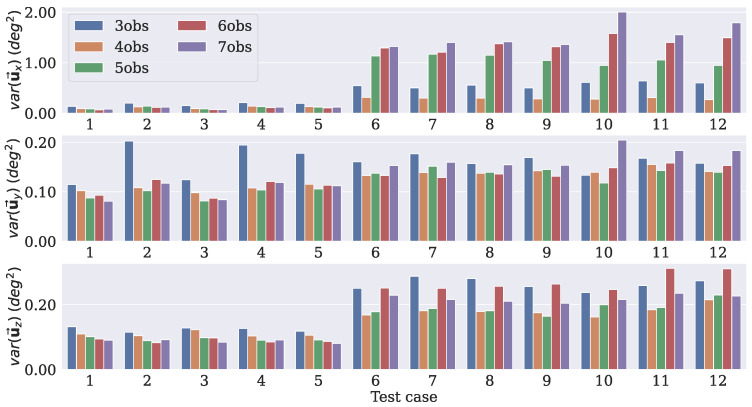
Uncertainties in the axes for each test case considering a dropout rate of 15%.

**Figure 6:**
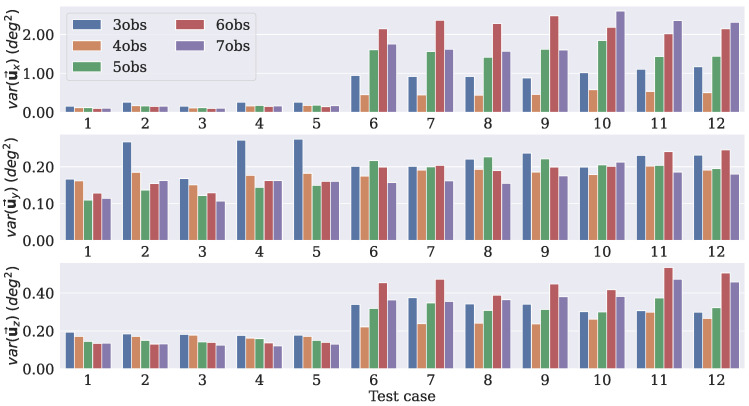
Uncertainties in the axes for each test case considering a dropout rate of 20%.

Since $\vec{u_{j}}$ is a representation of the difference rotation matrix $\Delta R_{j}$, by taking the diagonal entries of $C(\vec{u})$, it is possible to have the variances in the x, y, and z axes of the predicted DCMs with respect to $R_{\mathrm{avg}}$. This procedure was performed for each test case to measure the uncertainty of the rotation axes predicted by each model. The measured variances (in degrees${}^{2}$) for each dropout rate are displayed in Figure 4, Figure 5 and Figure 6.

The models with a dropout rate of 10% had lower variance levels in the three axes for the majority of the test cases. It is worth mentioning that these models were tested with the same dropout rate as that with which they were trained, that is, the models that were trained with a dropout rate of 10% were tested with the same rate, and so on. By doing that, it was possible to verify whether the number of observation vectors presented the same behavior independent of the dropout rate used. For the $\vec{u_{y}}$ and $\vec{u_{Z}}$ components, the values were similar, but the model that was trained using four observations had smaller variances for the $\vec{u_{X}}$ component compared to the others.

These results led to the choice of the model that was trained with four observation vectors as the best option. Since the experiments dealt with directional data, it was not possible to use the standard equations of the mean and variance to calculate the uncertainty. Nonetheless, the dropout procedure was still applied during the inference, so it generated different outputs for the same input. Therefore, it was still possible to use those predictions and the covariance approach to estimate the uncertainty.

**Figure 7:**
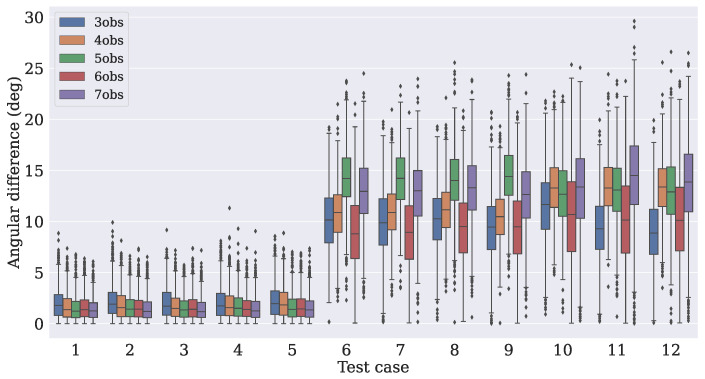
Dispersion of the absolute angular difference when using a dropout rate of 10%.

**Figure 8:**
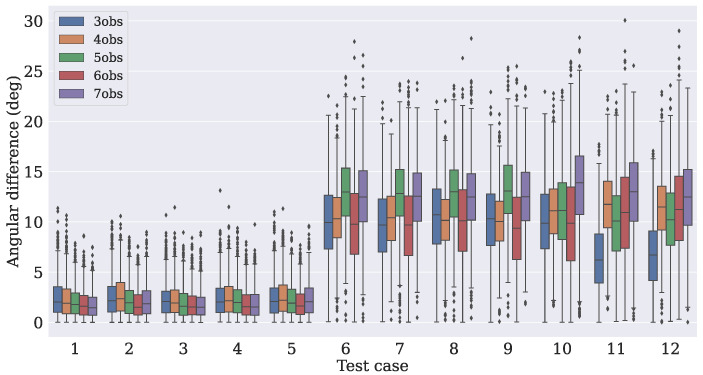
Dispersion of the absolute angular difference when using a dropout rate of 15%.

**Figure 9:**
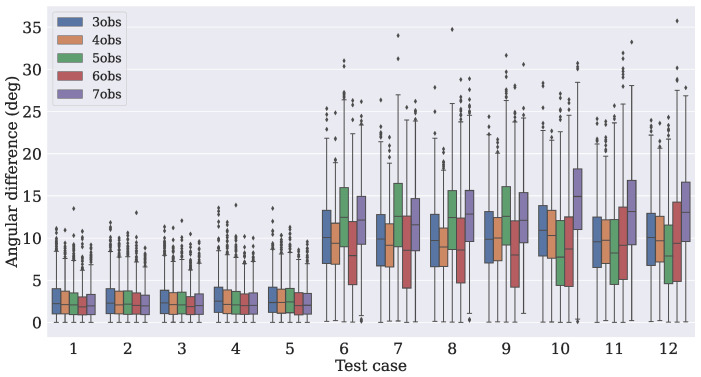
Dispersion of the absolute angular difference when using a dropout rate of 20%.

The distance between the true angle (extracted from Equation (7)) and the angles from the predicted attitude matrices $A_{\mathrm{pred}}$ was measured. For each test case, N predictions were taken, with their angles extracted using Equation (6). Figure 7, Figure 8 and Figure 9 show how the absolute angular differences (in degrees) were spread for the three dropout values. It can be noticed that the dispersion of the differences was narrower for the model that used four observation vectors.

**Figure 10:**
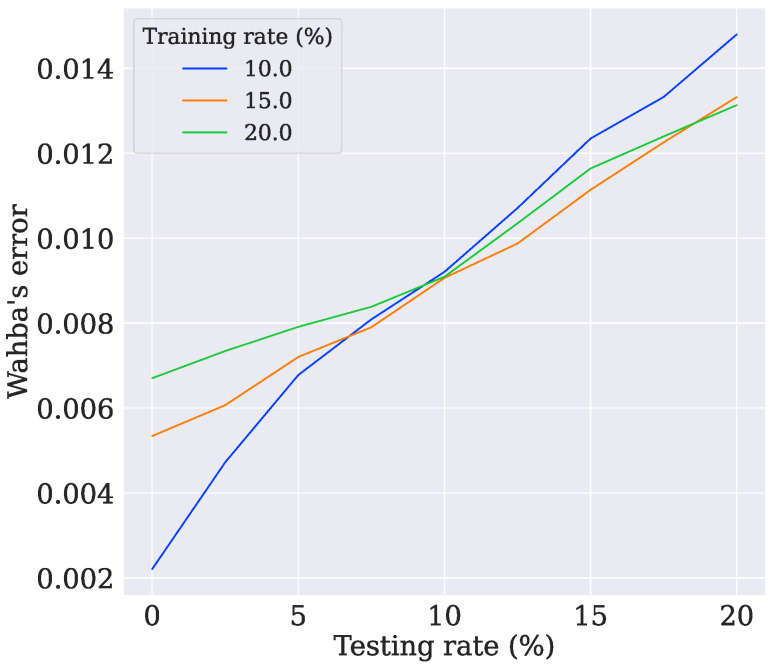
The evolution of Wahba’s error for test case 8.

**Figure 11:**
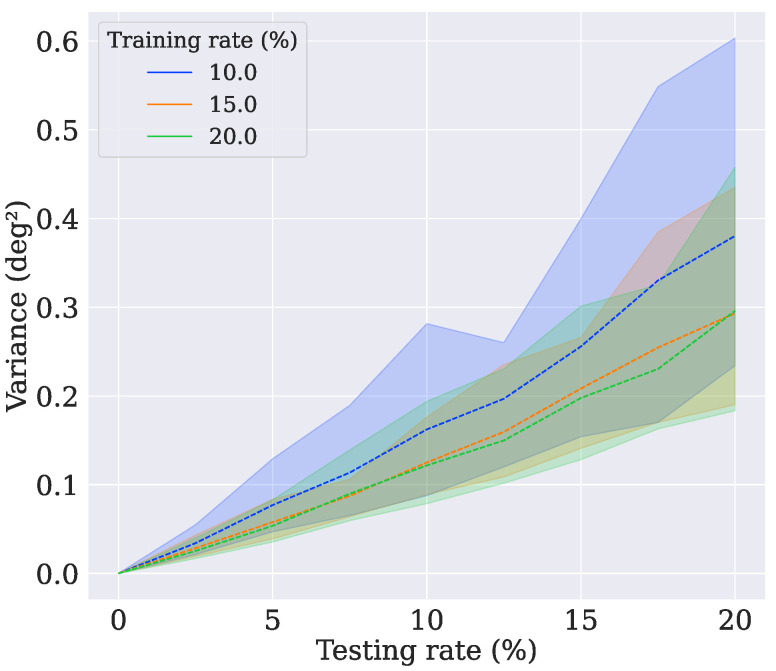
The evolution of the variance for test case 8.

It was verified that the model trained with four vectors had the best results. To better understand the models, two new tests were performed. The first one evaluated the behavior of the models when using another dropout rate during the testing. The other estimated how capable the models were of retaining knowledge when the number of switched-off neurons was varied. Wahba’s error and the variance of the B-Swish-4obs model were evaluated as they were previously, but the dropout rate was swept from 0% to 20% with steps of 2.5%. As shown in Figure 10, when the dropout rate increased, it was possible to notice that the model trained with 10% could not retain much knowledge compared with the other models, even though it achieved lower errors when the dropout layer was disabled (0%). Similarly, the model trained with 10% became more uncertain of its predictions as the testing dropout rate increased, as can be seen in Figure 11. The upper bound refers to the variance in the x-axis, the lower bound refers to the z-axis, and the dashed lines refer to the variance in the y-axis.

The results presented in Figure 10 and Figure 11 are only relevant if the model is running in an unstable environment, that is, it is expected that some neurons could “be lost” during inference; thus, the model has to be robust against the number of active neurons. To perform a comparison with the traditional algorithms, it was necessary to use the best scenario, that is, the B-Swish-4obs trained with a dropout rate of 10%, since it resulted in the lowest Wahba’s error when the dropout layers were disabled.

### 4.3. Comparison with Traditional Algorithms

To have confirmation that the chosen neural network model is the most appropriate one for the problem in question, a benchmark was carried out by using QUEST [39], an SVD-based method [40], the q-method, and ESOQ2. To do that, the twelve test cases defined above were used, but now, S samples were generated for each case and their respective measurement noises $N(0,\sigma_{i})$ were applied. For the tests, S=4000 was considered.

**Figure 12:**
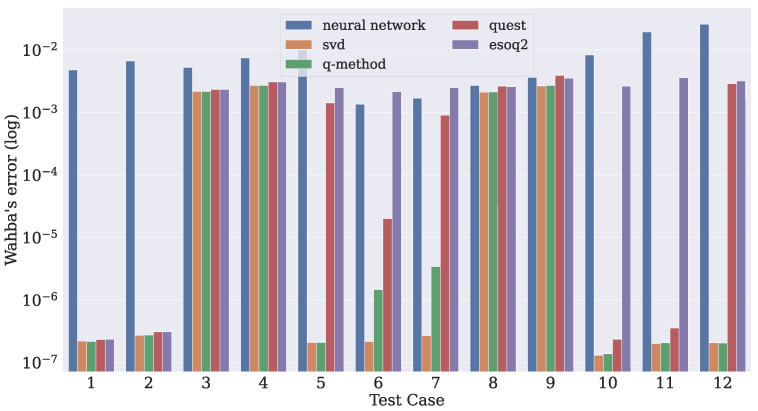
Comparison of the evaluation of Wahba’s error among some algorithms.

For each sample used in the algorithm, Wahba’s error was calculated by using Equation (1) with the value of $a_{i}$ given by each test case. After all samples had been evaluated, the mean of all errors $\sum_{j=1}^{S}L\left({\hat{R}}_{j}\right)$ was evaluated for each case. This procedure was executed for all algorithms. The dropout layers were disabled during inference since there was no need for evaluating the model uncertainty in this phase. The results are depicted in Figure 12 on a logarithmic scale for better visualization.

The SVD-based and q-method algorithms achieved the lowest errors, since they were the most robust. In some situations, such as in cases 3, 4, 8, and 9, all algorithms had similar results, since the inputs to the system had more significant associated measurement noise. In spite of the fact that the neural network performed worse in most cases, the predictions were more constant, that is, when considerable noise was introduced, the system error did not increase proportionally in the same way as in the other algorithms.

**Figure 13:**
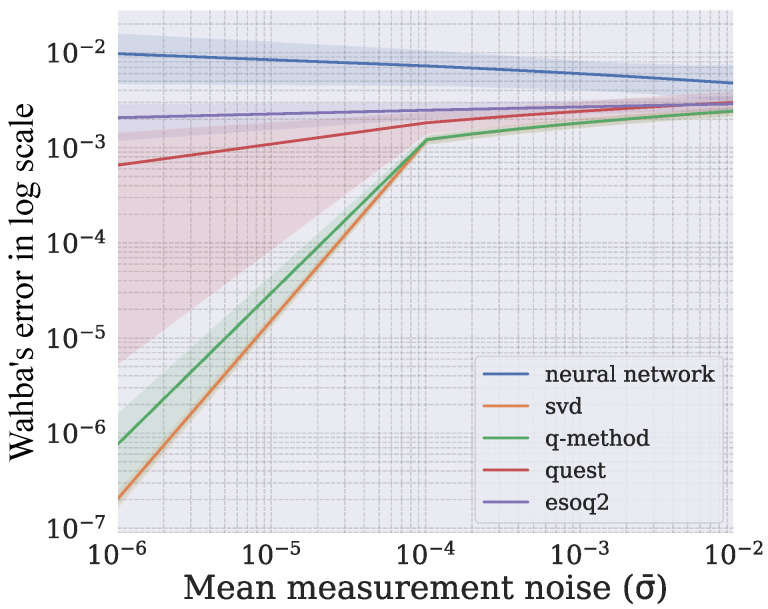
Impact of the average measurement noise on model performance.

The relationship between the measurement noise and Wahba’s error is presented in Figure 13. This analysis considered a weighted average of the measurement noises for each test case by using Equation (12). It is possible to notice that Wahba’s error remained constant in the neural network when the weighted average increased, unlike with the traditional algorithms. In addition, the presence of a single accurate observation vector (low-valued σ) in a test case was enough to prevent the conventional algorithm from performing poorly. Case number 10 is an example of this statement, since the traditional algorithms become less affected by the noise despite having two measurement noises with higher values.

## 5. Conclusions

This paper proposes a neural network model for the problem of static attitude determination based on an existing architecture called PointNet. Modifications to the system’s layers, input shapes, and output shapes were performed due to the poor results in preliminary tests. It was noticed that changing the ReLU activation function to Swish resulted in an improvement in the loss with the validation set. The most noticeable gain happened when the input used in the system was changed, which was probably because the network was unable to obtain meaningful information about how the pairs of observation and reference vectors were correlated. In addition, traditional algorithms use calculation techniques that may require more computational resources, such as SVD, eigenvectors, and eigenvalues. With the proposed method, after the neural network is trained on Earth (offline training), the embedded system will rely on pre-established weights; thus, it will only require the active calculation of multiplications and additions.

Using a star-tracking scenario during the training stage resulted in smaller values of Wahba’s error during the testing stage than those obtained when using the actual weights. This is interesting because it removes the need to store the actual weights $a_{i}$ in order to build the attitude profile matrix B. The error with the validation data indeed decreased when the number of measurement vectors increased. This did not reflect the actual behavior when the test case scenarios were run. This might be because the test cases used only up to three vectors, and the models that were trained using more observation vectors could not generalize very well for fewer vectors. However, for the same test cases, the variances of the predicted DCMs were not out of line around the y and z axes when measuring the uncertainty. On the other hand, they had significant differences for the x axis, especially for cases 6 to 12, where the input vectors had a boresight along the x axis.

While the chosen model could not surpass the traditional algorithms when the input vectors had more significant measurement noise associated with them, it did not suffer from this noise as the other algorithms did, achieving similar values of Wahba’s error; therefore, it is less sensitive to the input noise. As future work, it is suggested Bingham’s distribution concepts be incorporated into the model. As our output data are somehow a type of directional data, it may be possible to have a better estimation of the uncertainty, perhaps by reducing this value and obtaining some improvements in Wahba’s loss. Thus, the effective use and testing of the proposed solution in real situations need to be considered.

## Figures and Tables

**Table 1:** Wahba’s error for each model (the best result for each model is defined in bold).

Model	3obs			4obs			5obs			6obs			7obs		
**Dropout Rate (%)**	**10**	**15**	**20**	**10**	**15**	**20**	**10**	**15**	**20**	**10**	**15**	**20**	**10**	**15**	**20**
**Case**															
1	−2.03	−1.96	−1.90	−2.04	−2.00	−1.93	**−2.17**	−2.02	−1.99	−2.10	−2.08	−1.99	−2.14	−2.07	−1.97
2	−1.81	−1.75	−1.68	−1.86	−1.78	−1.73	−1.92	−1.82	−1.76	−1.86	−1.85	−1.76	**−1.95**	−1.80	−1.76
3	−2.00	−1.94	−1.90	−2.06	−1.98	−1.92	**−2.12**	−2.02	−1.97	−2.08	−2.05	−1.98	−2.11	−2.06	−1.97
4	−1.80	−1.74	−1.69	−1.86	−1.78	−1.74	**−1.92**	−1.83	−1.75	−1.85	−1.84	−1.75	−1.91	−1.79	−1.77
5	−1.82	−1.75	−1.69	−1.81	−1.72	−1.71	**−1.94**	−1.79	−1.78	−1.90	−1.88	−1.79	−1.93	−1.79	−1.74
6	−1.94	−1.87	−1.85	**−2.05**	−1.97	−1.91	−1.89	−1.87	−1.71	−1.75	−1.78	−1.73	−1.83	−1.77	−1.75
7	−1.77	−1.67	−1.65	**−1.87**	−1.78	−1.72	−1.70	−1.68	−1.53	−1.58	−1.61	−1.53	−1.65	−1.60	−1.57
8	−1.92	−1.85	−1.83	**−2.02**	−1.96	−1.87	−1.89	−1.86	−1.69	−1.75	−1.77	−1.74	−1.82	−1.76	−1.74
9	−1.75	−1.68	−1.65	**−1.85**	−1.78	−1.71	−1.71	−1.69	−1.52	−1.56	−1.60	−1.55	−1.65	−1.60	−1.56
10	**−1.95**	−1.92	−1.83	−1.88	−1.91	−1.88	−1.92	−1.90	−1.81	−1.73	−1.73	−1.76	−1.73	−1.70	−1.64
11	**−1.75**	−1.68	−1.65	−1.65	−1.63	−1.62	−1.67	−1.65	−1.65	−1.64	−1.56	−1.54	−1.54	−1.58	−1.54
12	−1.56	−1.53	−1.53	−1.54	−1.58	−1.57	−1.49	**−1.64**	−1.53	−1.37	−1.44	−1.46	−1.39	−1.46	−1.35

## Data Availability

Not applicable.
